# Supplementary material for: Thermal responses of dissolved organic matter under global change
Source: Nat Commun. 2024 Jan 17;15:576. doi: 10.1038/s41467-024-44813-2 (PMC10794202; doi:10.1038/s41467-024-44813-2)
Supplement: Supplementary file 1 — Supplementary Information [file 41467_2024_44813_MOESM1_ESM.pdf]

Supplementary Information for

Thermal response of dissolved organic matter under global change

Ang Hu[1], Kyoung-Soon Jang[2], Andrew J. Tanentzap[3], Wenqian Zhao[1], Jay T. Lennon[4], Jinfu Liu[1], Mingjia Li[1], James Stegen[5], Mira Choi[2], Yahai Lu[6], Xiaojuan Feng[7], Jianjun Wang[1,*]

[1] Key Laboratory of Lake and Watershed Science for Water Security, Nanjing Institute of Geography and Limnology, Chinese Academy of Sciences, Nanjing 210008, China

[2] Bio-Chemical Analysis Team, Korea Basic Science Institute, Cheongju 28119, South Korea

[3] Ecosystems and Global Change Group, Department of Plant Sciences, University of Cambridge, Cambridge CB2 3EA, United Kingdom

[4] Department of Biology, Indiana University, Bloomington IN 47405, USA

[5] Pacific Northwest National Laboratory, 902 Battelle Boulevard, P.O. Box 999, Richland, Washington 99352, USA

[6] College of Urban and Environmental Sciences, Peking University, Beijing 100871, China

[7] State Key Laboratory of Vegetation and Environmental Change, Institute of Botany, Chinese Academy of Sciences, Beijing 100093, China

[*] Correspondence: jjwang@niglas.ac.cn

The file includes:

Supplementary Figures (S1–S15)

Supplementary Figures

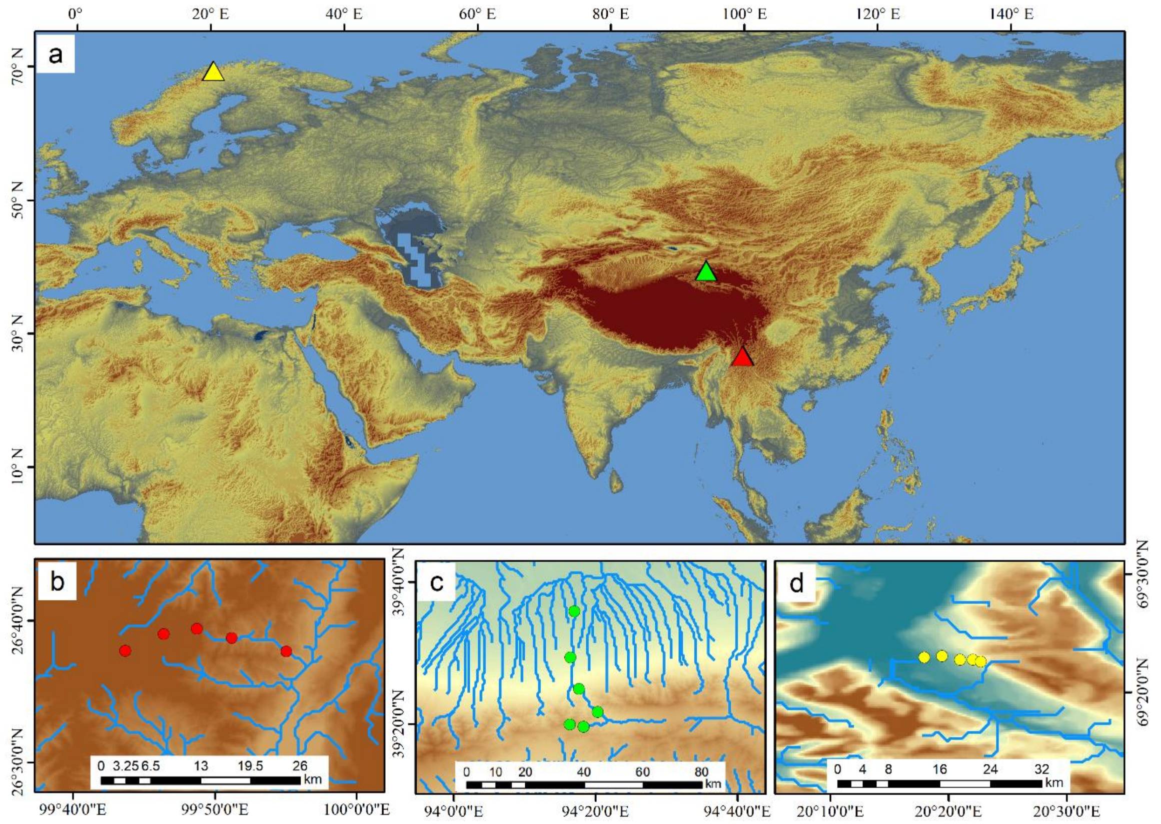

Figure S1. Study area on the three mountainsides across the contrasting climatic zones for field microcosm experiments (a). For the subtropical wet climate, we selected the Laojun Mountain in the southeastern margin of the Tibetan Plateau, China (red triangle). For the temperate arid climate, we selected the Dangjin Mountain in the northern Tibetan Plateau, China (green triangle). For the subarctic climate, we selected the Balggesvarri Mountain in Northern Europe, Norway (yellow triangle). On each mountainside, we selected five to six different elevations. The elevations were 3,822, 3,505, 2,915, 2,580 and 2,286 m a.s.l. on Laojun Mountain (red dots; b), 3,905, 3,477, 3,050, 2,670, 2,214 and 1,750 m a.s.l. on Dangjin Mountain (green dots; c), and 750, 550, 350, 170 and 20 m a.s.l. on Balggesvarri Mountain (yellow dots; d). Sources: GMTED2010 layer at 7.5 arc-sencond resolution (https://www.usgs.gov/) and GloRic version 1.0 layer at 15 arc-second resolution (https://www.hydrosheds.org/page/gloric).

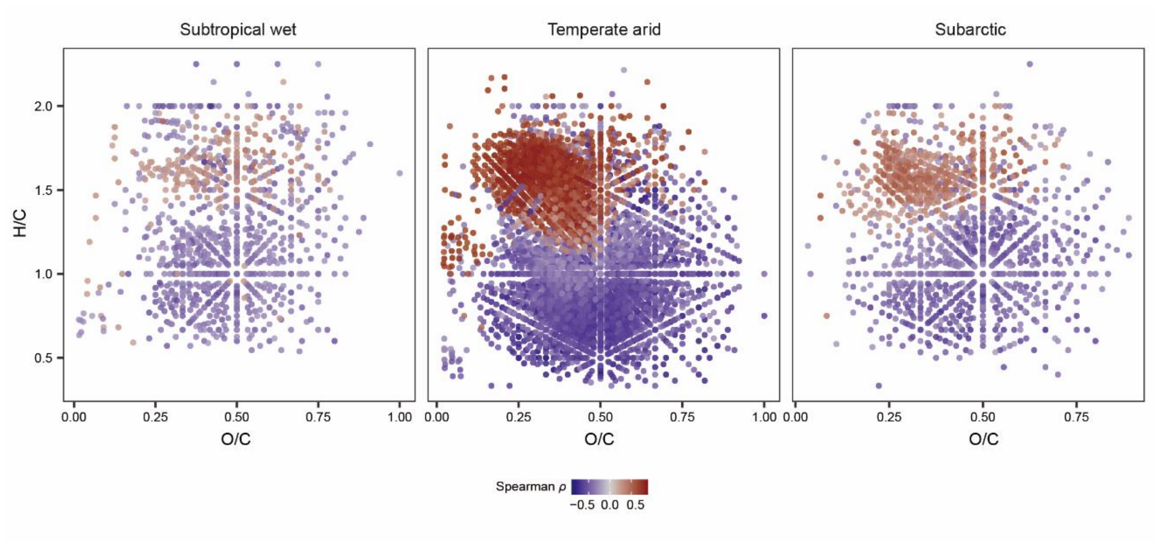

Figure S2. The distribution of DOM molecules with positive (red) or negative (blue) molecular thermal responses (MERs) in van Krevelen diagrams in the subtropical wet (Laojun Mountain, China), temperate arid (Dangjin Mountain, China) and subarctic (Balggesvarri Mountain, Norway) climate zones. MERs were quantified with the Spearman's correlation coefficient ρ between the relative abundance of each DOM molecule and water temperature. Each molecule is colored by its MER.

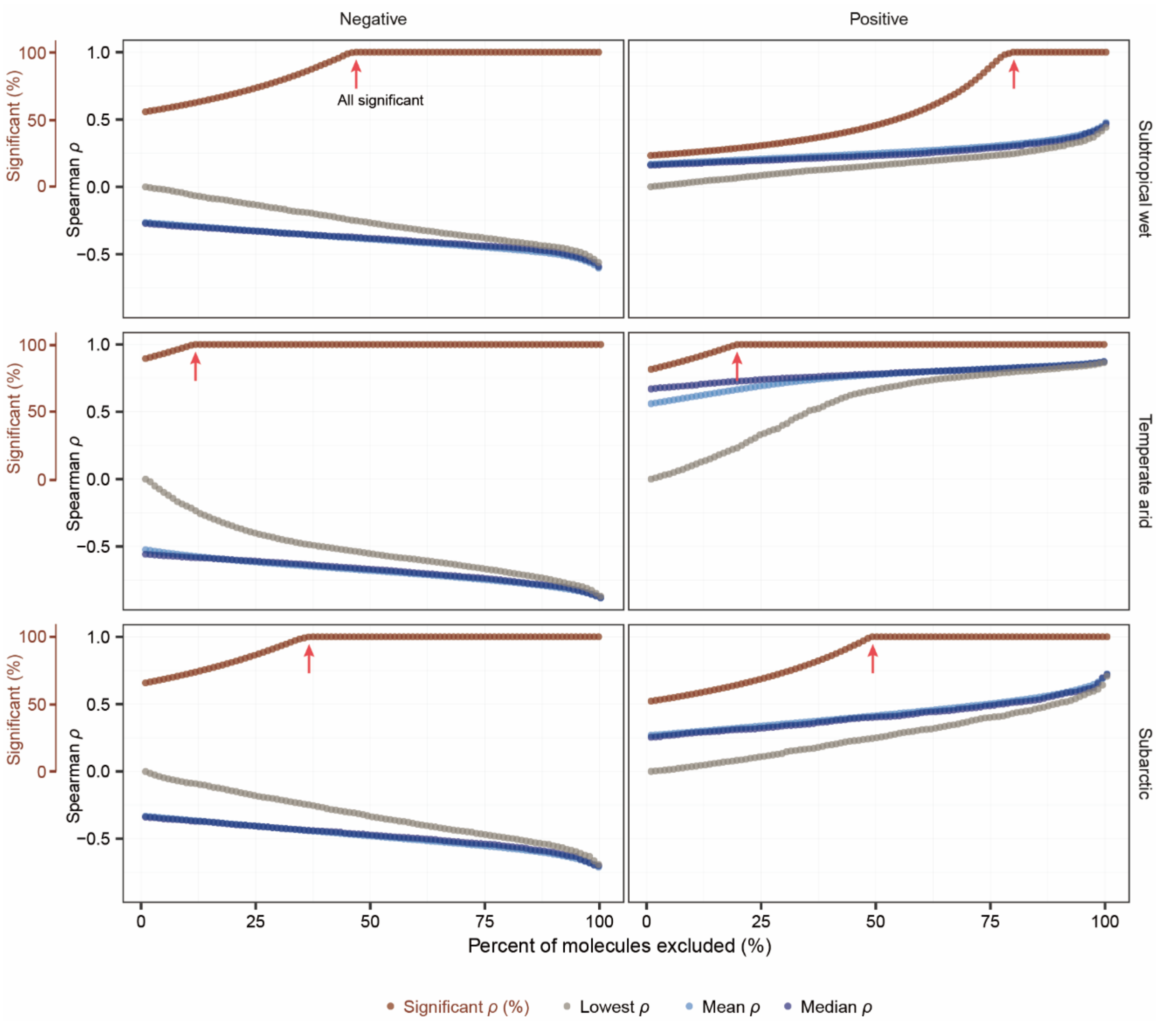

Figure S3. Molecular features along the gradient of the percentage of molecules removed. Features included the percentage of statistically significant molecular thermal responses (MERs, %), and the lowest, mean and median MERs. MERs were quantified with the Spearman's correlation coefficient ρ between the relative abundance of each DOM molecule and water temperature. We categorized all molecules into 100 bins (that is, 1% of molecules per bin) according to the magnitude of their positive or negative MERs. We sequentially removed the molecules from the bins with lower magnitude of MERs and then calculated molecular features of the remaining molecules.

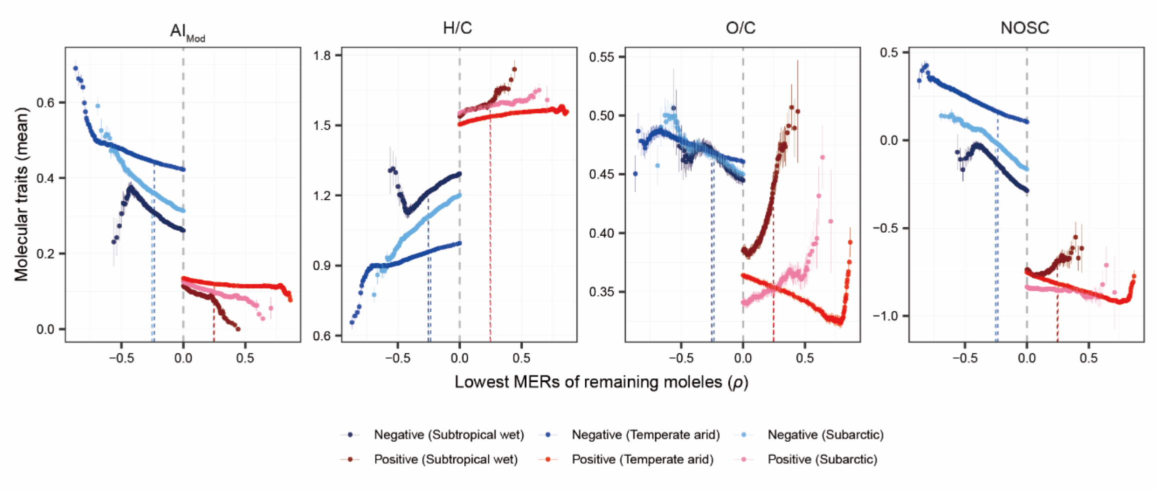

Figure S4. The gradual changes in molecular traits along the continuum of negative (blue) to positive (red) molecular thermal responses (MERs) in the subtropical wet (Laojun Mountain, China), temperate arid (Dangjin Mountain, China) and subarctic (Balggesvarri Mountain, Norway) climate zones. We considered molecular traits of modified aromaticity index (AI_{Mod}), H/C ratio, O/C ratio, and nominal oxidation state of carbon (NOSC). We categorized all molecules into 100 equal-sized bins (that is, 1% of molecules per bin) according to the magnitude of their positive or negative MERs. We sequentially removed the molecules from the bins with lower magnitude of MERs and generated 100 removal scenarios. The measurements of molecular traits were visualized by plotting against the lowest MERs of remaining molecules for each removal scenario. Blue and red dashed lines highlight the cutoff of 100% significantly negative and positive MERs for the remaining molecules, respectively. At a given removal scenario, we also considered other molecular features such as the percentage of significant responses (%), the percentage of molecules removed, and mean and median MERs (see Fig. S3). Data are presented as the means ± s.e averaged across molecules for each removal scenario. In the first removal scenarios, $n = 1{,}431$, 3,829 and 1,417 molecules for the warm-depleting molecules in the three climate zones, respectively; $n = 979$, 1,620 and 914 molecules for the warm-accumulating molecules in the three climate zones, respectively. These n numbers were then sequentially reduced by removal of one bin of molecules and not provided here considering hundreds of numbers.

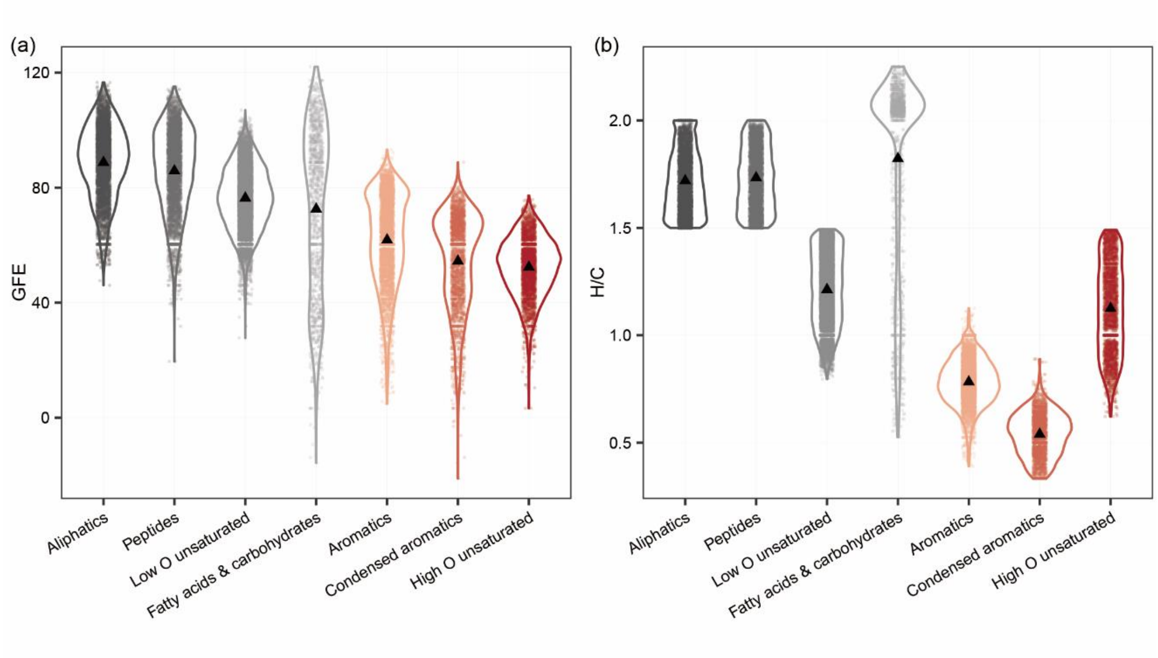

Figure S5. Violin plots of Gibbs free energy (GFE, a) and H/C ratio (b) across compound classes of DOM. Smaller dots are the values for individual molecules; minima and maxima are the top and bottom of the violin, and the black triangles show the mean values for all molecules within each compound class. Aliphatics, $n = 4{,}702$; peptides, $n = 4{,}161$; low O unsaturated, $n = 9{,}445$; fatty acids & carbohydrates, $n = 1{,}306$; aromatics, $n = 4{,}351$; condensed aromatics, $n = 2{,}422$; high O unsaturated, $n = 3{,}113$ molecules. The violin colors from grey to red represent compound classes with different GFE varying from high to low values.

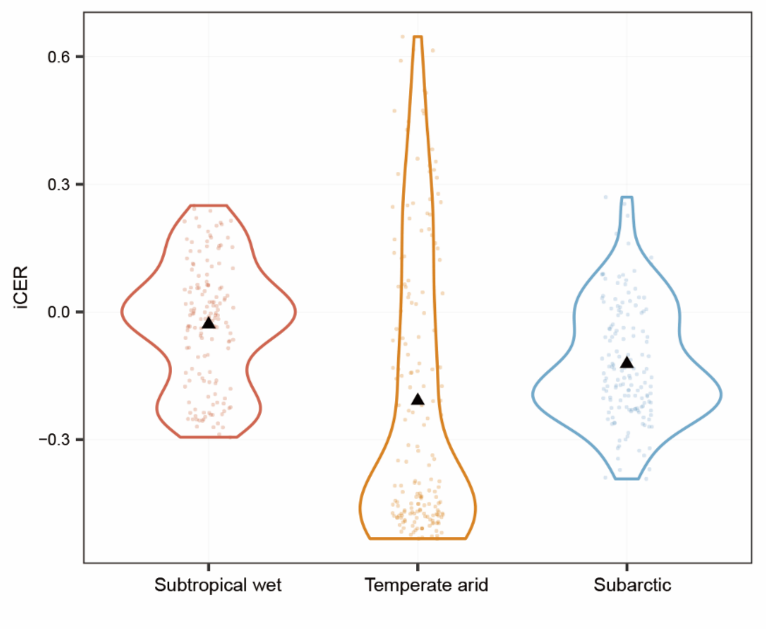

Figure S6. Violin plots of the indicator of compositional-level thermal responses (iCER) calculated with statistically significant ($P \leq 0.05$) molecular thermal responses (MERs) in the subtropical wet (Laojun Mountain, China), temperate arid (Dangjin Mountain, China) and subarctic (Balggesvarri Mountain, Norway) climate zones. Smaller dots are the iCER for individual samples; minima and maxima are the top and bottom of the violin, and the black triangles show the mean values for each region (i.e., climate zone). Subtropical wet, $n = 150$; temperate arid, $n = 180$; subarctic, $n = 150$ samples.

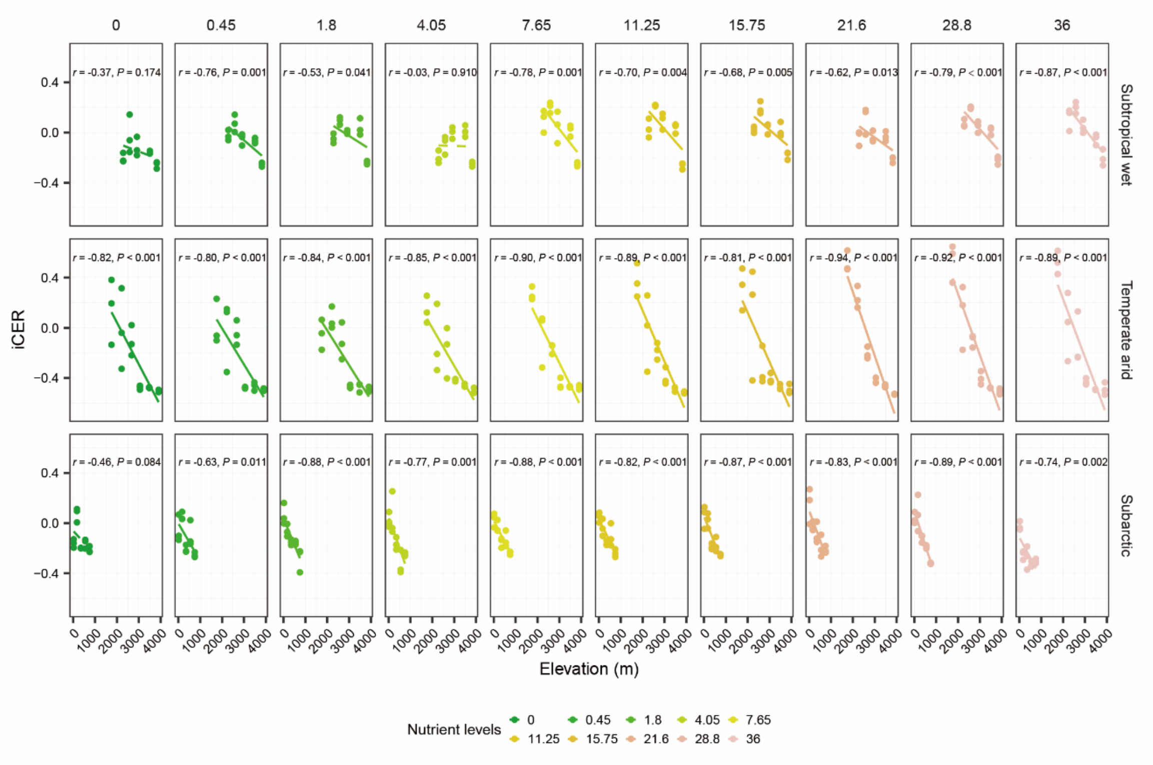

Figure S7. The elevational patterns in the indicator of compositional-level thermal responses (iCER) of DOM across 10 nutrient levels in the subtropical wet (Laojun Mountain, China), temperate arid (Dangjin Mountain, China) and subarctic (Balggesvarri Mountain, Norway) climate zones. The iCER values were calculated with statistically significant ($P \leq 0.05$) molecular thermal responses (MERs). We plotted iCER against elevation for each nutrient level and statistical significance of linear model fits with one-sided F-statistics is indicated by solid ($P \leq 0.05$) or dotted ($P > 0.05$) lines.

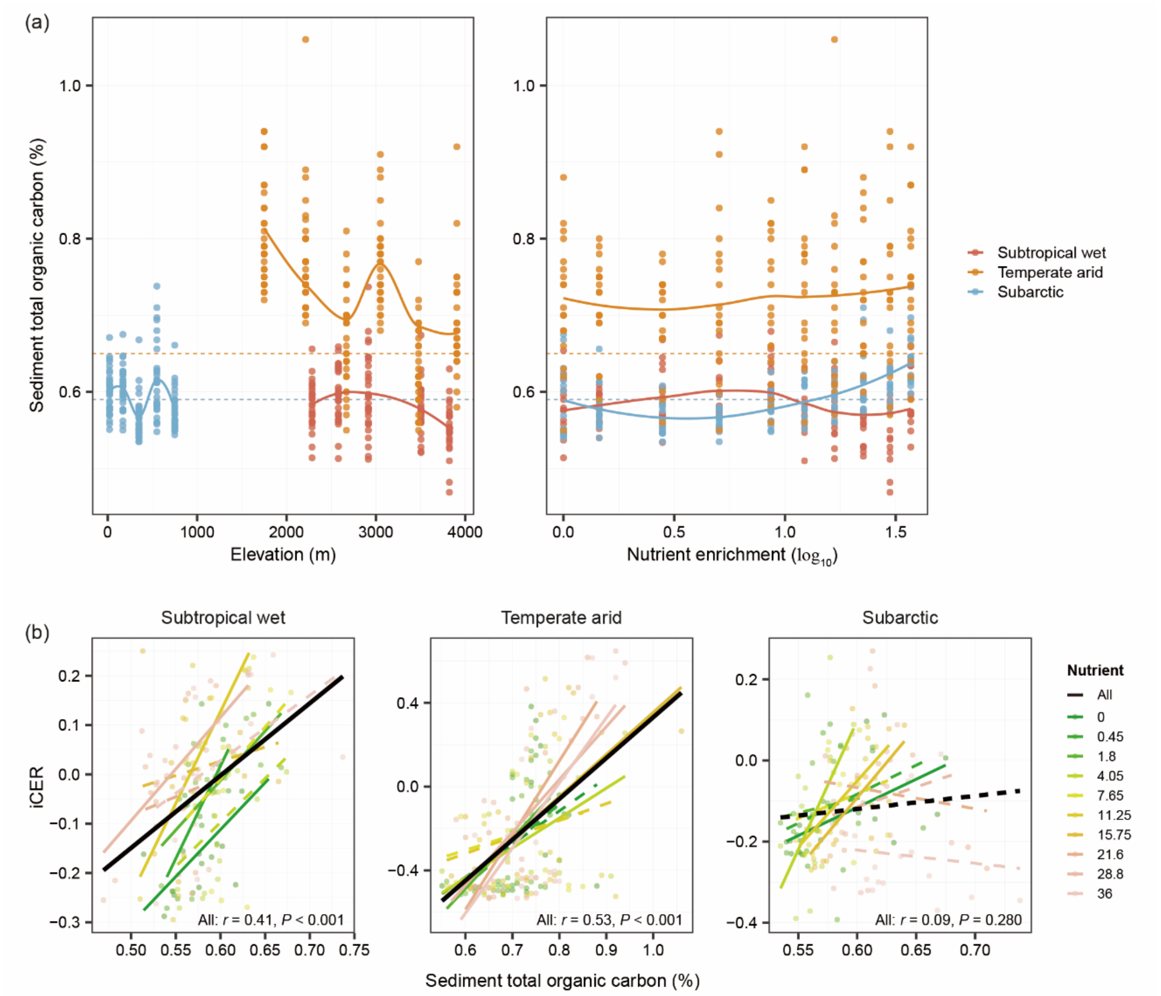

Figure S8. Total organic carbon (TOC) concentrations of sediments along the elevational (left panel) and nutrient (right panel) gradients (a), and their relationships with the indicator of compositional-level thermal responses (iCER, b) in the subtropical wet (Laojun Mountain, China), temperate arid (Dangjin Mountain, China) and subarctic (Balggesvarri Mountain, Norway) climate zones. The relationships in (a) were visualized with loess regression models. Blue dotted lines indicate the initial sediment TOC in the subtropical wet and subarctic climate zones, and orange dotted lines indicate the initial sediment TOC in the temperate arid climate zone. We plotted the iCER against sediment total organic carbon for all nutrients (black line) and each nutrient level (colored lines). The relationships in (b) are indicated by solid ($P \leq 0.05$) and dotted ($P > 0.05$) lines estimated using linear models with one-sided F-statistics.

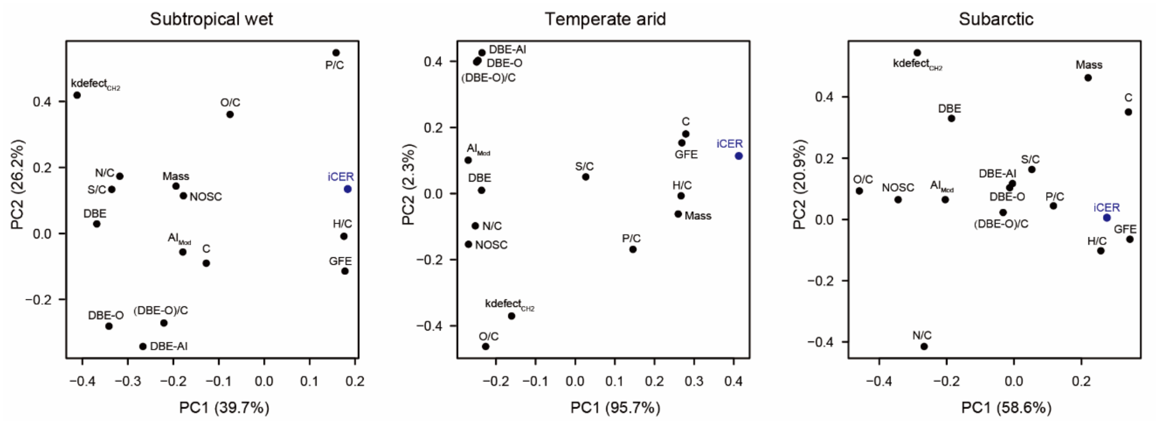

Figure S9. The principal component analysis (PCA) shows relationships among iCER and weighted means of formula-based DOM traits in the subtropical wet (Laojun Mountain, China), temperate arid (Dangjin Mountain, China) and subarctic (Balggesvarri Mountain, Norway) climate zones. The strong relationships between iCER and Gibbs free energy (GFE) were well supported by the results in both field and laboratory experiments shown in Fig. S10. We found the strongest correlations between iCER and almost all traits with two-sided Pearson *r* values of 0.90 to 0.99 ($P < 0.001$) in the temperate arid climate zone, which may lead to higher explained variance of the first principal component than the other two climate zones. Abbreviations of DOM traits are detailed in the Methods.

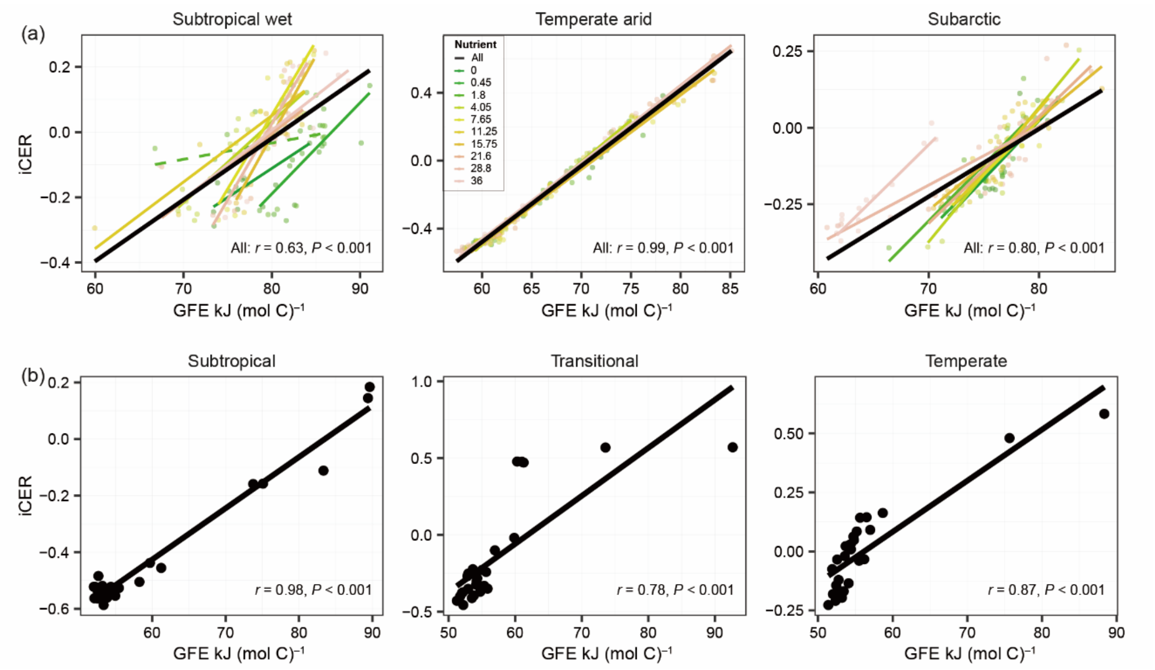

Figure 10. The changes in the indicator of compositional-level thermal responses (iCER) against weighted means of formula-based Gibbs free energy (GFE) in the field (a) and laboratory (b) experiments. In the field experiments, we showed their relationships for all nutrients (black line) and each nutrient level (colored lines) in the subtropical wet (Laojun Mountain, China), temperate arid (Dangjin Mountain, China) and subarctic (Balggesvarri Mountain, Norway) climate zones. In the laboratory experiments, we plotted iCER against GFE in the subtropical climate, temperate to subtropical transitional climate, and temperate climate zones. Statistical significance of linear model fits with one-sided F-statistics is indicated by solid ($P \leq 0.05$) or dotted ($P > 0.05$) lines.

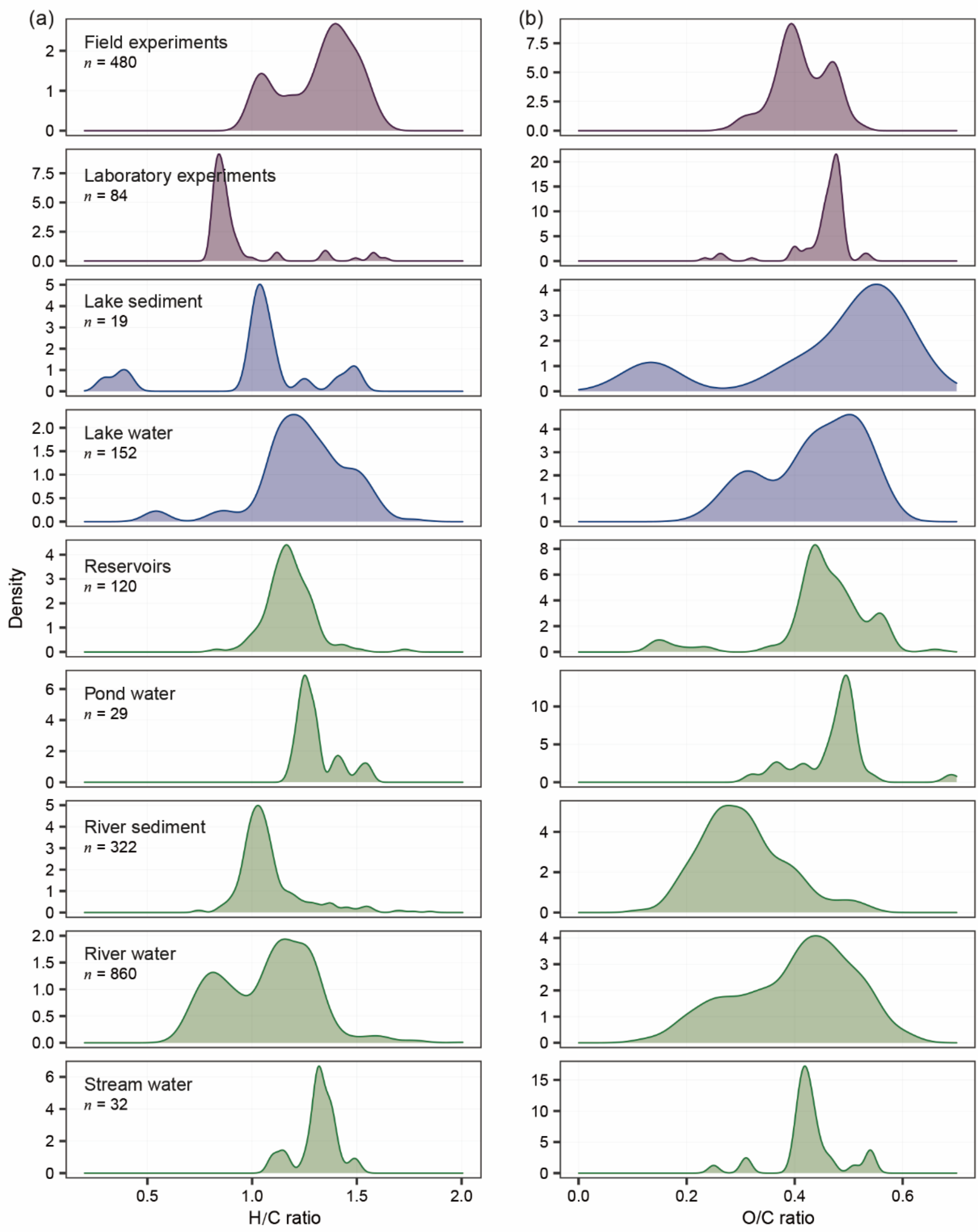

Figure S11. Density plots show the distribution of compositional-level H/C (a) and O/C (b) ratios of DOM from the field and laboratory experiments in this study, and also from main natural habitats of freshwaters (e.g., lake sediment, lake water, reservoirs, pond, river sediment, river water, and stream water). We synthesized the DOM characteristics derived from FT-ICR MS across the global natural freshwaters that are publicly available until June 2022. More details of global freshwater DOM are shown in Hu *et al*. (2023) [1]. *n* is the number of samples in each experiment or natural habitat.

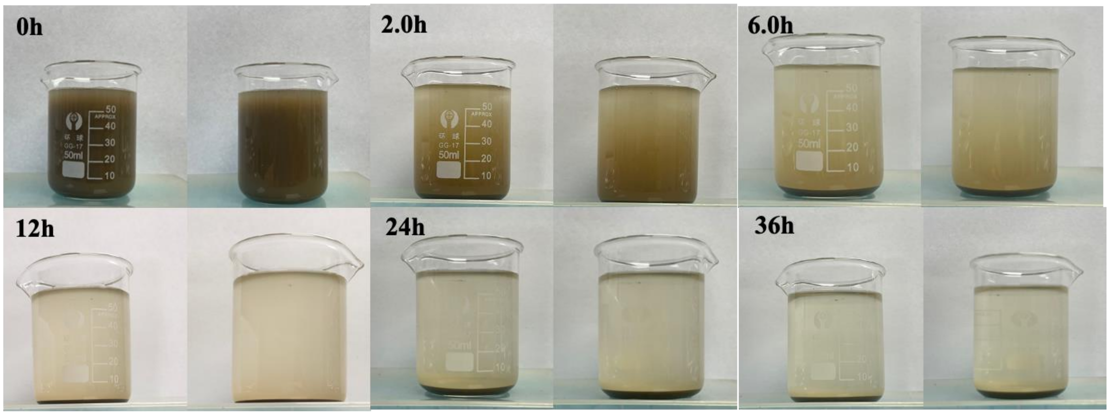

Figure S12. The pictures show the dynamics for sediments to settle down after they were added into microcosms and mixed with artificial lake water. We simulated the dynamics in beakers in laboratory with the same sediments and artificial lake water. It should be similar to the real field experimental conditions regarding the sediment settlement. We mixed the sediments with water is because we expected the sediments to be evenly distributed in the bottom of microcosms at the initial state of experiments. We found it would take at least one day for the sediments to settle down in the system.

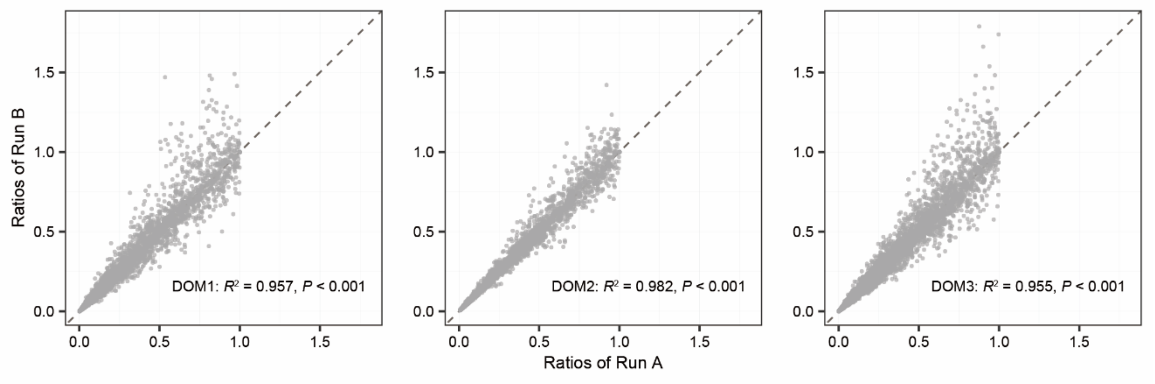

Figure S13. Ratios of peak intensities of Run A plotted against Run B for three DOM samples. Ratios of peak intensities were calculated as the ratio of the peak's intensity to the intensity of the base peak at each nominal *m*/*z* by following the method in Sleighter *et al*. (2012) [2]. We compared the relative peak intensities between the replicates (run A and run B) of DOM samples, and found there was a high reproducibility of replicate mass spectra of DOM analyzed by FT-ICR MS. The adjusted R^2 of linear models with one-sided F-statistics was obtained for the relationships between DOM replicates. Dashed line marks 1:1 relationship.

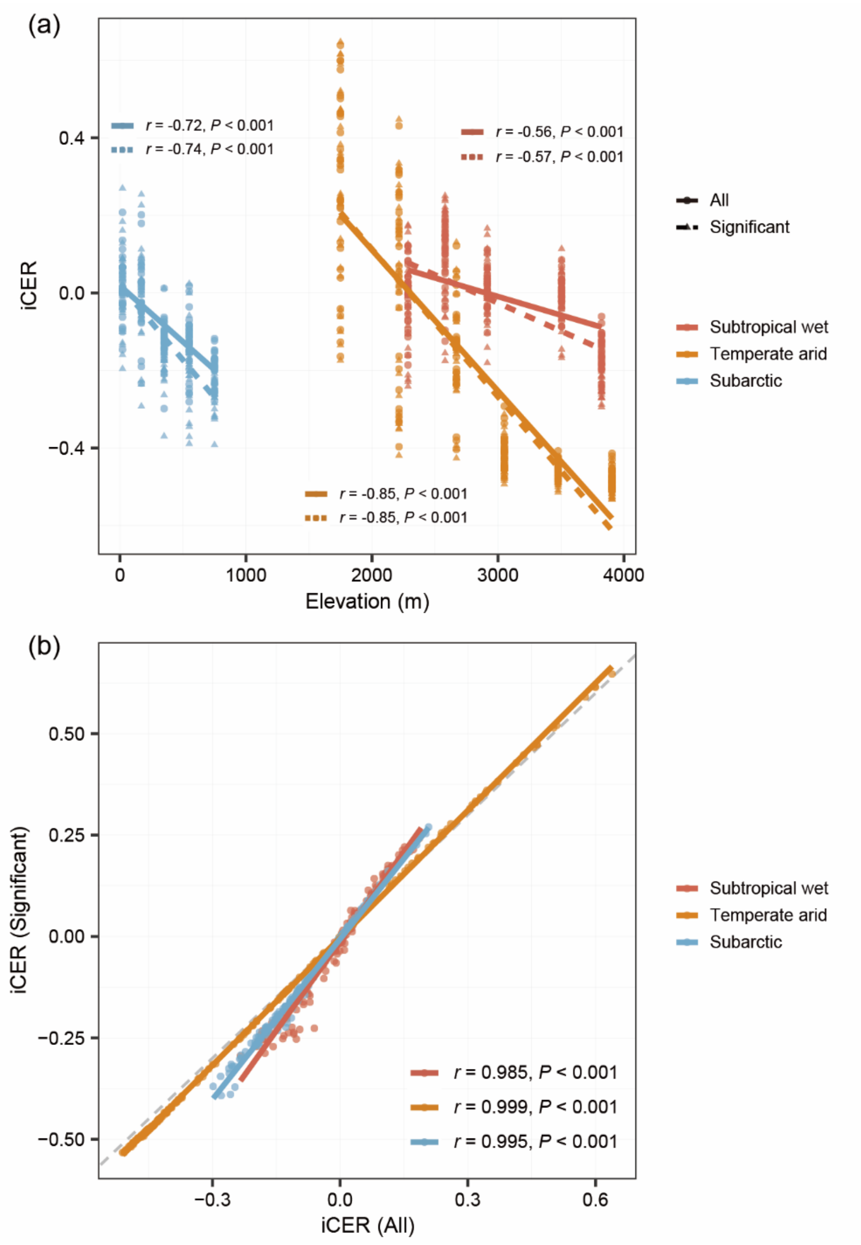

Figure S14. The elevational patterns in the indicator of compositional-level thermal responses (iCER) of DOM in the subtropical wet (Laojun Mountain, China), temperate arid (Dangjin Mountain, China) and subarctic (Balggesvarri Mountain, Norway) climate zones. (a) We plotted the iCERs, calculated using all molecules (solid lines) and significant ($P \leq 0.05$) molecular thermal response (MER) molecules (dashed lines), against elevation. (b) The relationships between the iCERs calculated using all molecules and significant MER molecules. The significance was examined using one-sided F-statistics.

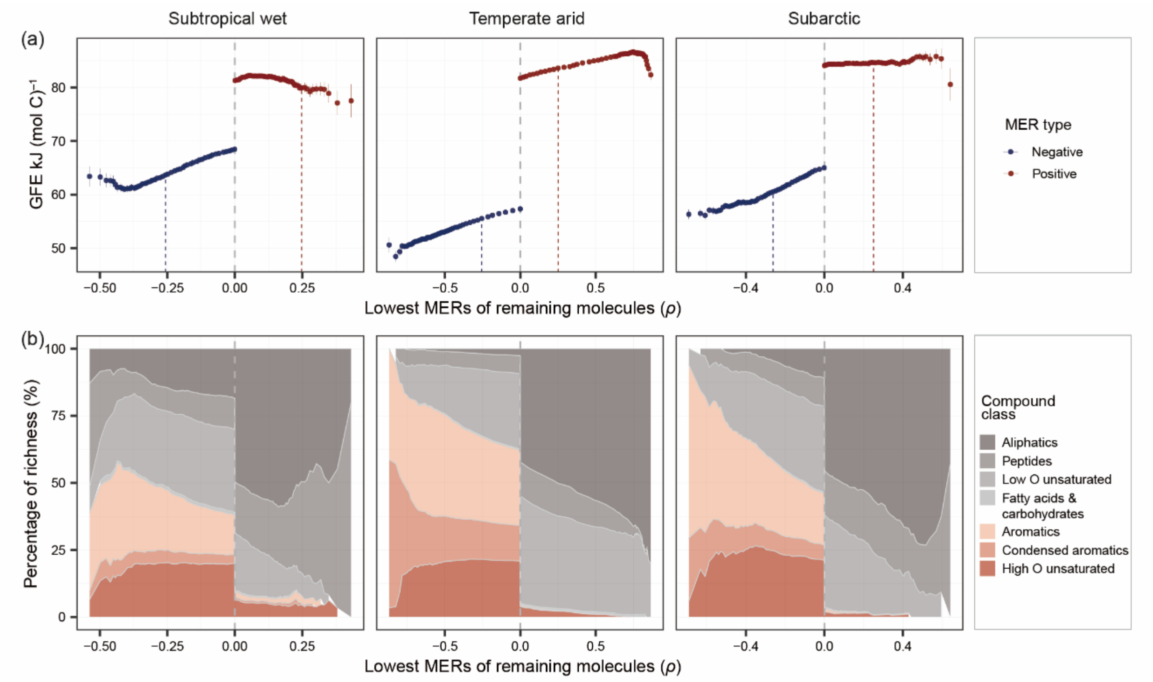

Figure S15. Gradual changes in molecular traits (Gibbs free energy, GFE; a) and the percentage of molecular richness (i.e., molecular peak number) for each compound class (b) along the continuum of negative (blue) to positive (red) MERs in the three climate zones. We used the bin size of 2% of all molecules to create the MER continuum, by categorizing all molecules into 50 equal-sized bins according to their magnitude of positive or negative MERs. Blue and red dashed lines in (a) highlight the cutoff of 100% significantly negative and positive MERs for the remaining molecules, respectively. Data in (a) are presented as the means ± s.e averaged across molecules for each removal scenario. In the first removal scenarios, n = 1,431, 3,829 and 1,417 molecules for the warm-depleting molecules in the three climate zones, respectively; n = 979, 1,620 and 914 molecules for the warm-accumulating molecules in the three zones, respectively. These n numbers were then sequentially reduced by removal of one bin of molecules and not provided here considering hundreds of numbers. The area colors from grey to red in (b) represent compound classes with different GFE varying from high to low.

References

1. Hu A, Han L, Lu X, Zhang G, Wang J. Global patterns and drivers of dissolved organic matter across Earth systems: Evidence from H/C and O/C ratios. *PREPRINT (Version 1) available at Research Square* doi:10.21203/rs.21203.rs-3324551/v3324551 (2023).

2. Sleighter RL, Hatcher PG. The application of electrospray ionization coupled to ultrahigh resolution mass spectrometry for the molecular characterization of natural organic matter. *J Mass Spectrom* **42**, 559-574 (2007).
