# Supplementary material for: Thermal responses of dissolved organic matter under global change
Source: Nat Commun. 2024 Jan 17;15:576. doi: 10.1038/s41467-024-44813-2 (PMC10794202; doi:10.1038/s41467-024-44813-2)
Supplement: Supplementary file 3 — Reporting Summary [file 41467_2024_44813_MOESM3_ESM.pdf]

nature portfolio

Corresponding author(s): Jianjun Wang

Last updated by author(s): Dec 29, 2023

Reporting Summary

Nature Portfolio wishes to improve the reproducibility of the work that we publish. This form provides structure for consistency and transparency in reporting. For further information on Nature Portfolio policies, see our Editorial Policies and the Editorial Policy Checklist.

Statistics

For all statistical analyses, confirm that the following items are present in the figure legend, table legend, main text, or Methods section.

n/a	Confirmed	
☐	☒	The exact sample size (*n*) for each experimental group/condition, given as a discrete number and unit of measurement
☐	☒	A statement on whether measurements were taken from distinct samples or whether the same sample was measured repeatedly
☐	☒	The statistical test(s) used AND whether they are one- or two-sided<br>*Only common tests should be described solely by name; describe more complex techniques in the Methods section.*
☐	☒	A description of all covariates tested
☐	☒	A description of any assumptions or corrections, such as tests of normality and adjustment for multiple comparisons
☐	☒	A full description of the statistical parameters including central tendency (e.g. means) or other basic estimates (e.g. regression coefficient) AND variation (e.g. standard deviation) or associated estimates of uncertainty (e.g. confidence intervals)
☐	☒	For null hypothesis testing, the test statistic (e.g. *F*, *t*, *r*) with confidence intervals, effect sizes, degrees of freedom and *P* value noted<br>*Give P values as exact values whenever suitable.*
☒	☐	For Bayesian analysis, information on the choice of priors and Markov chain Monte Carlo settings
☒	☐	For hierarchical and complex designs, identification of the appropriate level for tests and full reporting of outcomes
☐	☒	Estimates of effect sizes (e.g. Cohen's *d*, Pearson's *r*), indicating how they were calculated

Our web collection on statistics for biologists contains articles on many of the points above.

Software and code

Policy information about availability of computer code

Data collection	NA
Data analysis	All statistical analyses and plots were run in Formularity, R statistical software with its packages. Details of these softwares are available in the material and methods.

For manuscripts utilizing custom algorithms or software that are central to the research but not yet described in published literature, software must be made available to editors and reviewers. We strongly encourage code deposition in a community repository (e.g. GitHub). See the Nature Portfolio guidelines for submitting code & software for further information.

Data

Policy information about availability of data

All manuscripts must include a data availability statement. This statement should provide the following information, where applicable:

- Accession codes, unique identifiers, or web links for publicly available datasets
- A description of any restrictions on data availability
- For clinical datasets or third party data, please ensure that the statement adheres to our policy

The DOM and associated meta data, and the thermal response data for the field and laboratory experiments have been deposited at https://doi.org/10.5281/zenodo.8435631. The environmental data of Taihu Lake are accessible via request from Taihu Laboratory for Lake Ecosystem Research. The other information is available from the corresponding author upon reasonable request.

The scripts and example data in the procedures of calculating iCER indicator are available at http://github.com/jianjunwang/iDOM and https://doi.org/10.5281/zenodo.8435631.

Human research participants

Policy information about studies involving human research participants and Sex and Gender in Research.

Reporting on sex and gender	NA
Population characteristics	NA
Recruitment	NA
Ethics oversight	NA

Note that full information on the approval of the study protocol must also be provided in the manuscript.

Field-specific reporting

Please select the one below that is the best fit for your research. If you are not sure, read the appropriate sections before making your selection.

☐ Life sciences ☐ Behavioural & social sciences ☒ Ecological, evolutionary & environmental sciences

For a reference copy of the document with all sections, see nature.com/documents/nr-reporting-summary-flat.pdf

Ecological, evolutionary & environmental sciences study design

All studies must disclose on these points even when the disclosure is negative.

Study description	Macroecological field experiments performed on three Eurasian mountainsides in contrasting climates, by considering two environmental gradients for aquatic microcosms: Temperature and nutrient. We also conducted laboratory incubation experiments under warming treatments. We examined sediment DOM composition and the responses of DOM molecules to temperature change.
Research sample	There are 480 microcosms in field experiments, including five to six elevations from three mountains. For each elevation, ten nutrient levels with three replicates. There are 84 microcosms in laboratory experiments, including three climate zones. For each climate zone, seven temperature treatments with four replicates.
Sampling strategy	For field experiments, we selected locations with five to six different elevations on each mountainside. The elevations were 3,822, 3,505, 2,915, 2,580 and 2,286 m a.s.l. on Laojun Mountain in China, 3,905, 3,477, 3,050, 2,670, 2,214 and 1,750 m a.s.l. on Dangjin Mountain in China, and 750, 550, 350, 170 and 20 m a.s.l. on Balggesvarri Mountain in Norway. At each elevation, we established 30 aquatic microcosms (1.5 L bottle) composed of 15 g of sterilised lake sediment and 1.2 L of sterilised artificial lake water, which included one of ten nutrient levels of 0, 0.45, 1.80, 4.05, 7.65, 11.25, 15.75, 21.60, 28.80 and 36.00 mg N L-1 of KNO3.<br>For laboratory experiments, we established aquatic microcosms with the common sterilized Taihu Lake sediments, but with different microbial communities inoculated from lake sediments across three contrasting climate zones, and incubated them under a temperature gradient of 5, 10, 15, 20, 25, 30, and 35 °C. These three sediments from an unnamed lake in southern China (22.53194 N; 107.77140 E), Daputang Lake in mid-eastern China (32.08694 N; 118.92170 E), and Dongshan Lake in northeastern China (49.57444 N; 128.50330 E) in July-August 2021.
Data collection	For field experiments, we recorded data in situ on each mountain and tested the samples in lab. For laboratory experiments, we tested the samples in lab. More details could be found in the material and methods. The field trip was carried out led by Jianjun Wang.
Timing and spatial scale	The comparative field microcosm experiments were conducted on Laojun Mountain in the southeastern margin of the Tibetan Plateau in China (26.6959 N; 99.7759 E) in September-October 2013, on Dangjin Mountain in the northern Tibetan Plateau in China (39.4203 N; 94.2950 E) in September-October 2018, and on Balggesvarri Mountain in Northern Europe in Norway (69.3809 N; 20.3483 E) in July 2013. The laboratory experiments were conducted in September 2021.
Data exclusions	NA
Reproducibility	All details in field setups, laboratory analyses, and data analyses described in the material and methods.
Randomization	Samples were reordered for sample handling and FT-ICR MS to rule out potential biases.
Blinding	The sample order is independent among the testing of meta data and DOM analysis. The technicians are essentially having no information about the sample order or the relationships among the samples.

Did the study involve field work? ☒ Yes ☐ No

Field work, collection and transport

Field conditions	In the Laojun Mountain region, mean annual temperatures (MAT) and mean annual precipitations (MAP) ranged from 4.2 to 12.9 °C and 1,005 to 1,020 mm, respectively. In the Dangjin Mountain region, MAT and MAP ranged from -3.4 to 7.2 °C and 31 to 84 mm, respectively. In the Balggesvarri Mountain region, MAT and MAP ranged from -2.9 to 0.7 °C and 539 to 597 mm, respectively.
Location	The locations were 3,822, 3,505, 2,915, 2,580 and 2,286 m a.s.l. on Laojun Mountain in the southeastern margin of the Tibetan Plateau in China (26.6959 N; 99.7759 E), 3,905, 3,477, 3,050, 2,670, 2,214 and 1,750 m a.s.l. on Dangjin Mountain in the northern Tibetan Plateau in China (39.4203 N; 94.2950 E), and 750, 550, 350, 170 and 20 m a.s.l. on Balggesvarri Mountain in Northern Europe in Norway (69.3809 N; 20.3483 E).
Access & import/export	This is open field trip and no specific permission is required in respective of academic studies carried out by Nanjing Institute of Geography and Limnology (Chinese Academy of Sciences), and Department of Geosciences and Geography (University of Helsinki).
Disturbance	No specific disturbance was caused due to the small spatial scale needed for field experiments.

Reporting for specific materials, systems and methods

We require information from authors about some types of materials, experimental systems and methods used in many studies. Here, indicate whether each material, system or method listed is relevant to your study. If you are not sure if a list item applies to your research, read the appropriate section before selecting a response.

Materials & experimental systems

n/a	Involved in the study	
☒	☐	Antibodies
☒	☐	Eukaryotic cell lines
☒	☐	Palaeontology and archaeology
☒	☐	Animals and other organisms
☒	☐	Clinical data
☒	☐	Dual use research of concern

Methods

n/a	Involved in the study	
☒	☐	ChIP-seq
☒	☐	Flow cytometry
☒	☐	MRI-based neuroimaging
